# Evaluation of the Hepatocellular Carcinoma Predictive Scores PAGE-B and mPAGE-B among Brazilian Patients with Chronic Hepatitis B Virus Infection

**DOI:** 10.3390/v14091968

**Published:** 2022-09-05

**Authors:** Ana Caroline Ferreira da Silva, Marlone Cunha-Silva, Tiago Sevá-Pereira, Daniel F. Mazo

**Affiliations:** 1 Division of Gastroenterology (Gastrocentro), Department of Internal Medicine, School of Medical Sciences of University of Campinas (UNICAMP), Campinas 13083-878, SP, Brazil; 2 Division of Clinical Gastroenterology and Hepatology, Department of Gastroenterology, University of São Paulo School of Medicine (FMUSP), Sao Paulo 05403-900, SP, Brazil

**Keywords:** hepatitis B virus, hepatocellular carcinoma, risk factors

## Abstract

Hepatitis B virus (HBV) is intrinsically oncogenic and related to hepatocellular carcinoma (HCC). Predictive scores of HCC have been developed but have been poorly studied in admixed populations. Therefore, we aimed to evaluate the performance of PAGE-B and mPAGE-B scores for HCC prediction in HBV Brazilian patients and factors related to HCC occurrence. This is a retrospective study that evaluated patients followed at a tertiary university center. A total of 224 patients were included, with a median follow-up period of 9 years. The mean age at HBV diagnosis was 38.71 ± 14.19 years, predominantly males (66.1%). The cumulative incidence of HCC at 3, 5, and 7 years was 0.993%, 2.70%, and 5.25%, respectively, being related in the univariate logistic regression analysis to male sex (*p* = 0.0461), older age (*p* = 0.0001), cirrhosis at HBV diagnosis (*p* < 0.0001), and higher values of PAGE-B and mPAGE-B scores (*p* = 0.0002 and *p* < 0.0001, respectively). Older age, male sex, and cirrhosis at HBV diagnosis were independently associated with HCC occurrence. The AUROCs of PAGE-B and mPAGE-B were 0.7906 and 0.7904, respectively, with no differences between them (*p* = 0.9767). In conclusion, both PAGE-B and mPAGE-B showed a correct prediction of HCC above 70% in this cohort.

## 1. Introduction

Chronic hepatitis B virus (HBV) infection is a global public health problem. In 2019, it was estimated that 296 million individuals were living with this chronic liver disease, which accounted for about 820,000 deaths, mainly related to cirrhosis and hepatocellular carcinoma (HCC) [1]. Epidemiology data in Latin American countries is scarce and fragmented. However, it is believed that 7 to 12 million people have chronic HBV infection [2]. In Brazil, the prevalence of HBV infection in 2019 was approximately 6.3 per 100,000 inhabitants, and, from 2000 to 2019, hepatitis B was associated with 16,722 deaths [3].

In addition to causing liver parenchyma damage in a noncytopathic manner through hepatocellular necrosis, inflammation, and fibrosis, HBV is intrinsically oncogenic due to its characteristic of integrating into the human genome [4,5,6]. If untreated, chronic HBV infection is associated with a lifetime risk of HCC and liver-related mortality of 15% in women and 40% to 50% in men [7]. Factors that seem to influence these outcomes are viral-related (HBV viral load, genotype, mutations), host-related (age, gender, genetics, immune status), and environmental [8].

The rate of HCC development in untreated HBV subjects is estimated to be less than 1% in noncirrhotic patients and 2% to 5% in cirrhotic patients [9]. Although it does not eliminate the risk, long-term HBV antiviral treatment is related to a decrease in the incidence of HCC [10,11]. The cumulative incidence of HCC in 5 years among those on HBV antiviral therapy was 0.5% to 6.9% in subjects without cirrhosis, 4.5 to 21.6% in patients with compensated cirrhosis, and 46.5% in those with decompensated liver disease [12]. Therefore, HCC surveillance with abdominal ultrasound ± serum alpha-fetoprotein measurement every 6 months is recommended for HBV patients at higher risk [13,14,15].

However, to the already known difficulties in implementing HCC surveillance in clinical practice [16], the COVID-19 pandemic has brought additional challenges to proper HCC screening [17,18]. In this way, developing HCC predictor scores may help prioritize higher-risk patients. Several scoring systems have been proposed to quantify HCC risk, and the first developed in non-Asian patients was PAGE-B [19]. PAGE-B was developed in 2016 to predict the development of HCC in 5 years in Caucasian patients undergoing HBV treatment, composed by age, sex, and platelet count. This score had a c-index of 0.82, and the 5-year HCC cumulative incidence according to the classification into low, intermediate, and high risk was 0%, 3%, and 17%, respectively [19]. Subsequently, Kim et al. (2018) added the level of serum albumin, creating the mPAGE-B score. The authors described an area under the receiver-operating characteristic curve (AUROC) of 0.82 in the prediction of HCC in 5 years in Asians, with better performance than other evaluated scores: CU-HCC, GAG-HCC, REACH-B, THRI, and PAGE-B [20]. However, only one study evaluated PAGE-B [21] and none with m-PAGE-B in admixed populations, such as the Brazilian population.

In this context, this study describes the cumulative incidence of HCC, its associated factors, and the performance of PAGE-B and mPAGE-B in HCC prediction in HBV patients from a public tertiary university center in southeastern Brazil.

## 2. Materials and Methods

### 2.1. Clinical Design and Patients’ Selection

This is an observational, retrospective, single-center study carried out by reviewing the medical records of patients with chronic hepatitis B followed at the Viral Hepatitis Outpatient Clinic of the Division of Gastroenterology (Gastrocentro) of the University of Campinas (UNICAMP) from January 2000 to January 2021. The inclusion criteria were HBV monoinfected patients with hepatitis B surface antigen (HBsAg) positive for at least 6 months at any stage of infection. Exclusion criteria were other diagnosed chronic liver diseases (except previous significant use of alcohol), use of prophylaxis for HBV reactivation, HCC diagnosis in less than 6 months of follow-up, or incomplete data related to the variables studied. Study data collection was performed between September and November 2021.

### 2.2. Variables

Gender, age at diagnosis of HBV infection, hepatitis B e antigen (HBeAg) and HBsAg serology at the beginning and end of follow-up, treatment for hepatitis B during the follow-up (interferon or nucleos(t)ide analogs), and HCC were documented. The presence of cirrhosis was evaluated at HBV diagnosis and at the end of the medical records evaluation. Metabolic comorbidities (diabetes mellitus, dyslipidemia, systemic arterial hypertension, chronic kidney disease) and significant alcohol use were evaluated at any time during the follow-up. The period of follow-up at the service until January 2021, liver transplantation, HCC diagnosis, or death were registered.

The variables definition criteria used were:

• Type 2 diabetes mellitus (T2D): fasting blood glucose and/or glycated hemoglobin above 126 mg/dL and 6.5%, respectively, confirmed in a second measurement or use of medications for glucose control;
• Dyslipidemia: high-density lipoprotein (HDL) < 40 mg/dL in men, HDL < 50 mg/dL in women, low-density lipoprotein (LDL) > 160 mg/dL, triglycerides > 150 mg/dL or use of statin/fibrate;
• Systemic arterial hypertension (SAH): systolic blood pressure ≥ 140 mmHg and/or diastolic blood pressure ≥ 90 mmHg in at least two appointments or use of medication to treat SAH;
• Chronic kidney disease (CKD): estimated creatinine clearance ≤ 60 mL/min for at least 3 months or presence of kidney transplantation;
• Significant alcohol use: >20 g/day for women or >30 g/day for men [22];
• Cirrhosis: liver histology showing Metavir grade 4 fibrosis [23] or liver ultrasound with morphology suggestive of cirrhosis [24];
• HCC: histology or a three-phase liver imaging test (computed tomography or magnetic resonance imaging) with the observation of a hyper-uptake lesion in the arterial phase with rapid washout of the contrast in the portal or equilibrium phases [13,14,15].

### 2.3. Assessment of Risk Scores for HCC

Two 5-year predictor scores for HCC development were applied, PAGE-B and mPAGE-B [19,20]. PAGE-B is calculated by the sum of points attributed to platelet count/mm^3^ (≥200,000: 0 points; 100,000–199,999: 6 points; <100,000: 9 points); age (16–29 years: 0 points; 30–39 years: 2 points; 40–49 years: 4 points; 50–59 years: 6 points; 60–69 years: 8 points; ≥70 years: 10 points), and gender (female: 0 points; male: 6 points). Low-risk patients are defined by a score of ≤9 points, intermediate risk by 10–17 points, and high risk if ≥18 points [19]. mPAGE-B is calculated by the sum of points attributed to platelet count/mm^3^ (≥200,000: 0 points; 200,000–250,000: 2 points; 150,000–200,000: 3 points; 100,000–150,000: 4 points; <100,000: 5 points); age (<30 years: 0 points; 30–39 years: 3 points; 40–49 years: 5 points; 50–59 years: 7 points; 60–69 years: 9 points; ≥70 years: 11 points); gender (female: 0 points; male: 2 points), and level of serum albumin in g/dL (≥4.0: 0 points; 3.5–4.0: 1 point; 3–3.5: 2 points; <3: 3 points). Low risk is defined by a score of ≤8 points, intermediate risk by 9–12 points, and high risk if ≥13 points [20]. The scores were evaluated in two time points, whenever possible: at the diagnosis of HBV infection (initial PAGE-B and initial mPAGE-B), and 5 years before the development of HCC in those who developed this outcome.

### 2.4. Ethical Considerations

The University of Campinas Ethics Committee approved this study (number 4,960,167). The protocol was conducted following the ethical guidelines of the 2013 World Medical Association Declaration of Helsinki [25]. An informed consent form was applied to patients who attended regular medical consultations during the study’s data collection period.

### 2.5. Statistical Analysis

Frequency tables of the categorical variables with absolute frequency (*n*) and percentage (%) values and descriptive statistics of the numerical variables, with mean values, standard deviation (SD), minimum, maximum, and median values, were created. The chi-square test or Fisher’s exact test was used when necessary to compare proportions. To compare continuous measures between two groups, the Mann–Whitney test was applied. Logistic regression analysis was performed whenever possible. A stepwise variable selection approach was used in the multivariate logistic regression analysis. The hazard ratio (HR) of HCC development and 95% confidence interval (CI) were calculated.

The HCC cumulative incidence was assessed, as well as the cumulative probability of HCC stratified by baseline PAGE-B and mPAGE-B risk scores. The Log-rank test was used to compare the accumulated probabilities of HCC stratified by risk. The performance of the scores in the HCC prediction was analyzed through sensitivity, specificity, positive predictive value (PPV), negative predictive value (NPV), positive likelihood ratio (PLR), and negative likelihood ratio (NLR). Receiver-operating characteristic (ROC) curve graphs were drawn to assess the discrimination of the scores, and the AUROC was evaluated, with a CI of 95%. A probability value of <0.05 was considered significant. The Statistical Analysis System (SAS) for Windows software package, version 9.4 (SAS Institute Inc., 2002–2008, Cary, NC, USA) and R Core Team (R Foundation for Statistical Computing, 2021, Vienna, Austria) were used for the statistical analyses by the Statistics Service at the School of Medical Sciences of the University of Campinas.

## 3. Results

### 3.1. Patients’ Characteristics

**Figure 1:**
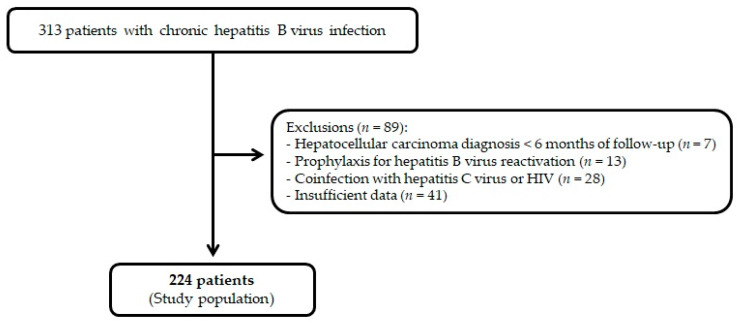
Flowchart of study population enrollment.

A total of 313 medical charts of patients diagnosed with HBV were evaluated, and, after exclusion criteria, 224 patients were included in the study (Figure 1).

The mean age at diagnosis was 38.71 ± 14.19 years, with a predominance of males (66.1%), with a median follow-up at the service of 108 (1–392) months (9 years). The most prevalent comorbidity was SAH (40.6%), and, at the beginning of the follow-up, 45 patients (45/205; 21.9%) had cirrhosis. Of the population studied, 30 had a history of significant alcohol use, 22 received liver transplantation, and 39 died during the study period. The minority had HBeAg positive at diagnosis (32.1%), and 14.7% of patients had HBsAg seroclearance at follow-up, with 123/224 (54.9%) receiving HBV treatment during follow-up. For treated patients, HBeAg and HBsAg seroclearance occurred in 32 and 16 patients, respectively. At the end of follow-up, 32 patients had cirrhosis and 11 patients were diagnosed with HCC. Among those patients without HBV treatment during follow-up, HBeAg and HBsAg seroclearance occurred in 8 and 17 patients, respectively. Twenty-three patients had cirrhosis and 4 patients had HCC. The characteristics of the study population are shown in Table 1.

### 3.2. HCC Occurrence and Associated Factors

During the study evaluation period, 15 (6.7%) patients were diagnosed with HCC. The mean and median length of time from initial outpatient follow-up until HCC diagnosis was 103.13 ± 78.99 months and 66 (11–273) months, respectively. The factors associated with HCC occurrence are presented in Table 2. There was a male predominance in HCC occurrence (93.3%, *p* = 0.0210), and no patient had previous significant alcohol intake. The mean age at diagnosis of HBV infection was higher in those with HCC (50.0 ± 12.58 vs. 37.89 ± 13.98 years, respectively, *p* < 0.0021). Higher initial PAGE-B and mPAGE-B scores and the presence of cirrhosis at HBV diagnosis and at the end of the follow-up (*p* < 0.0001) were also associated with the occurrence of HCC. There was no difference between HCC occurrence among patients regarding the presence of T2D, SAH, dyslipidemia, or CKD.

Univariate logistic regression was performed to assess the study population’s risk factors for developing HCC (Table 3). It was observed that males were 7.879 more likely to develop HCC (95%CI: 1.036–59.933, *p* = 0.0461). The presence of cirrhosis at baseline increased HCC risk by 21.531-fold (95%CI: 6.585–70.401, *p* < 0.0001). When higher, the PAGE-B and mPAGE-B values were also associated with HCC occurrence during follow-up (*p* = 0.0002 and *p* < 0.0001, respectively). In multivariate logistic regression analyses, age at HBV diagnosis (HR: 1.085; 95%CI: 1.034–1.149; *p* = 0.0009), male sex (HR: 8.298; 95%CI: 1.018–67.630; *p* = 0.0481), and cirrhosis at HBV diagnosis (HR: 11.720; 95%CI: 3.346–41.052; *p* = 0.0001) were independently related to the development of HCC.

### 3.3. Cumulative Incidence of HCC

**Figure 2:**
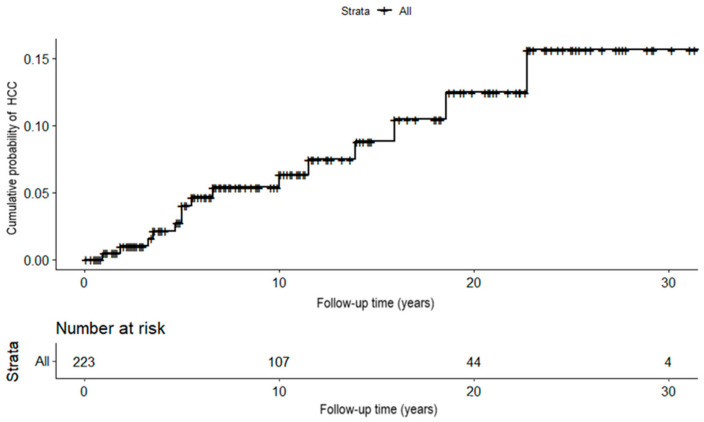
Cumulative incidence of hepatocellular carcinoma. HCC: hepatocellular carcinoma.

**Figure 3:**
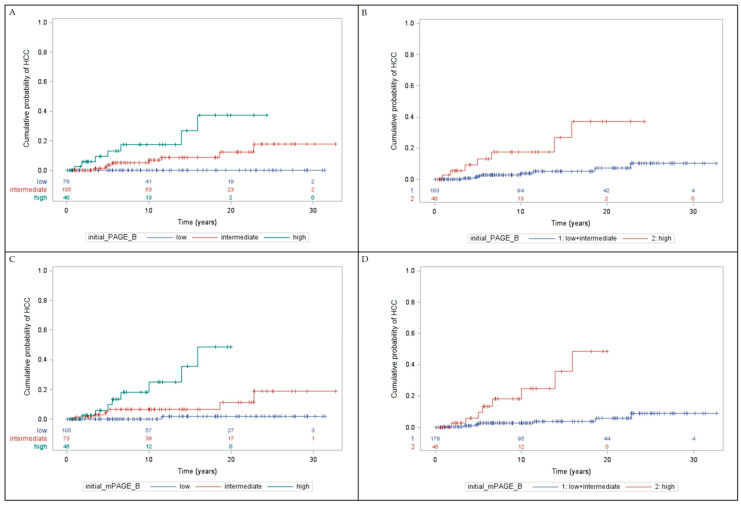
Cumulative probability of hepatocellular carcinoma stratified by the scores. (**A**): Initial PAGE-B; (**B**): initial PAGE-B (1 = low + intermediate risk, 2 = high risk)—Log rank *p* = 0.0001; (**C**): initial mPAGE-B—Log rank *p* < 0.0001; (**D**): initial mPAGE-B (1 = low + intermediate risk, 2 = high risk)—Log rank *p* < 0.0001. HCC: hepatocellular carcinoma.

The cumulative incidence of HCC at 3, 5, and 7 years was 0.993%, 2.70%, and 5.25%, respectively (Figure 2). In Figure 3 and Table 4, the cumulative probabilities of HCC are described based on the assessed risk scores. Both PAGE-B and mPAGE-B showed good discrimination between low- and intermediate- vs. high-risk groups over time (*p* = 0.0001 and *p* < 0.0001, respectively).

### 3.4. Performance of HCC Risk Scores

In the study population, according to PAGE-B stratification, 78 (34.8%) patients were classified as low-risk, 106 (47.3%) as intermediate-risk, and 40 (17.9%) as high-risk. Regarding mPAGE-B, the stratification in low-, intermediate-, and high-risk groups was 105 (46.9%), 74 (33.0%), and 45 (20.1%), respectively (Table 1). In the initial risk prediction analysis, patients who did not have HCC had median values of PAGE-B and mPAGE-B (12 and 9, respectively) lower than the median values of those patients who had HCC (17 and 13, respectively; *p* = 0.0002).

**Figure 4:**
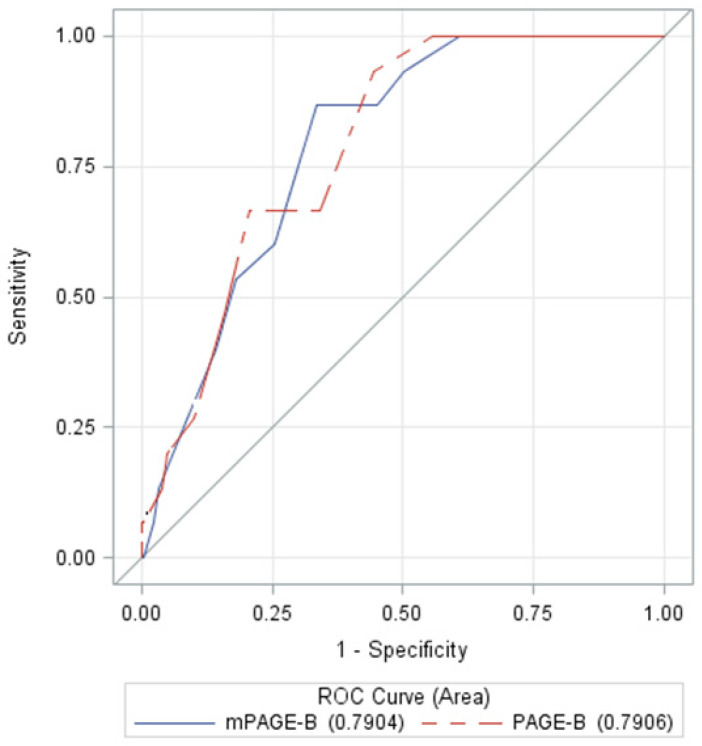
ROC curves of the initial PAGE-B and mPAGE-B scores (*p* = 0.9767). ROC: receiver operating characteristic.

Table 5 describes the results regarding the performance of the initial scores and Figure 4 presents the ROC curves. Both PAGE-B and mPAGE-B had relevant AUROCs of 0.7906 and 0.7904, respectively. There was no difference between the two scores regarding HCC prediction (*p* = 0.9767). The cutoff point for optimizing the probability of developing HCC was >14 for PAGE-B and >10.5 for mPAGE-B. PAGE-B and mPAGE-B showed high NPV of 99.14% and 98.58%, respectively.

**Figure 5:**
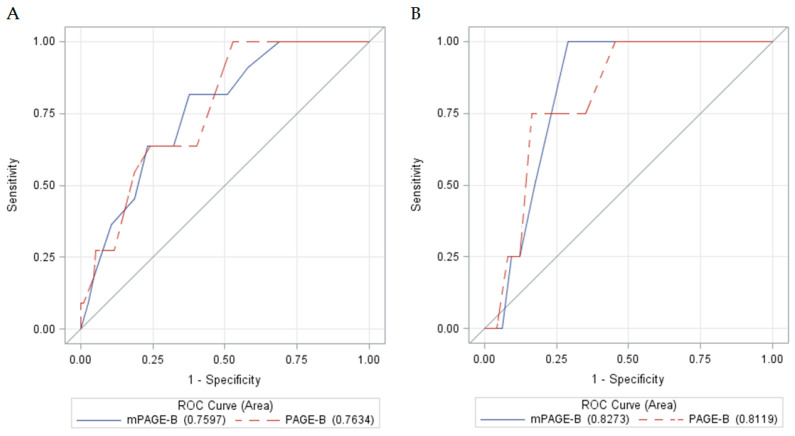
ROC curves of the initial PAGE-B and mPAGE-B scores stratified by HBV treatment during follow-up. (**A**): Patients that received HBV treatment (*p* = 0.9451); (**B**): patients without treatment during follow-up (*p* = 0.8138). HBV: hepatitis B virus; ROC: receiver-operating characteristic.

The performance of the scores was also evaluated in relation to HBV treatment during follow-up, as shown in Table 6 and Figure 5. In patients who received treatment, the AUROCs of PAGE-B and mPAGE-B were 0.7634 and 0.7597, respectively (*p* = 0.9451); in patients without HBV therapy, the AUROCs were 0.8119 and 0.8273, respectively (*p* = 0.8138).

Only one patient initially classified as low-risk by mPAGE-B developed HCC (11.5 years later), but when the score was recalculated 5 years before neoplasia occurrence, the risk was stratified as high. Assessing risk scores using the calculation 5 years before HCC diagnosis, the median values were higher in the group that developed HCC. In the PAGE-B evaluation, the median values in the groups that developed and did not develop HCC were 17.5 (12–24) and 12 (0–22), respectively (*p* = 0.0002). In the mPAGE-B evaluation, the median values in the groups that developed and did not develop HCC were 13 (10–17) and 9 (0–18), respectively (*p* = 0.0002). In the Cox univariate logistic regression analysis using the calculated scores 5 years before HCC occurrence, both scores significantly predicted HCC risk. For PAGE-B: HR: 1.335, 95%CI: 1.149–1.552, *p* = 0.0002, and for mPAGE-B: HR: 1.452, 95%CI: 1.212–1.741, *p* < 0.0001.

## 4. Discussion

Our study population of HBV subjects followed at a tertiary outpatient clinic consisted mainly of men, with a mean age of 38.71 years, and almost a quarter of them had cirrhosis at HBV diagnosis. HBeAg at baseline was present in 32.1%, its seroclearance occurred in 56.5% during the follow-up, and HBsAg seroclearance occurred in 14.7%. The most prevalent comorbidity was SAH, and 40.6% of the patients had cirrhosis at the end of the study evaluation. The prevalence of HCC in this population was 6.7% at a median follow-up time of 9 years, and its occurrence in the univariate logistic regression analysis was related to men, older age at HVB diagnosis, higher initial PAGE-B and mPAGE-B scores, and the presence of cirrhosis at HBV diagnosis. Older age, male gender, and cirrhosis at HBV diagnosis were independently associated with HCC development. The PAGE-B and mPAGE-B scores had AUROCs above 0.79 in the prediction of HCC in these patients, with no differences between them. In addition, both scores showed high negative predictive values.

Worldwide, regardless of endemicity, chronic hepatitis B prevalence is higher in males, and this factor is related to prognosis, such as the increased risk of HCC, cirrhosis, and death [5,7,26,27]. This is probably due to the greater replication of HBV in men and may reflect higher-risk sexual behavior and/or hormonal phenomena [27,28]. The present study population profile consisted of mostly men around 40 years old, which is in line with larger cohorts, such as those from the Korean National Health Insurance Service database composed of 317,856 patients [29]. The authors reported a global HCC prevalence of 5.93% after a median follow-up of 8.5 years. Despite having almost the same follow-up time, the occurrence of HCC was slightly lower than in the present study. Including fewer patients with cirrhosis than our study population (10.1% vs. 21.9%), a known risk factor for HCC, could have impacted HCC prevalence over time [29,30,31].

Older age was an independent risk factor for HCC in the present study, as previously described [19,20,32]. The presence of HBeAg at HBV diagnosis, also in line with a previous analysis by Yuen et al. (2009) [32], had no impact on HCC occurrence in the present population, neither did HBeAg and HBsAg seroclearance. Under antiviral treatment, HBV serology might not be as relevant in predicting HCC, which is reflected in HCC risk assessment models in patients on antiviral therapy that do not include viral load, transaminases, or HBeAg status [33,34].

Despite the lack of association in the present study, metabolic syndrome and its components may be related to an increased risk of HCC in HBV patients [29,31,35,36]. However, this finding is not universal across studies, as recently reported by Choe et al. (2021), evaluating 1,504,880 HBV-infected Korean adults from a regular national HCC screening program [37].

HCC is one of the most feared complications of chronic hepatitis B due to its high impact on morbidity and mortality. Therefore, much effort has been made to identify and validate its predictors [5,19,38,39]. Several scores were created and validated over time, but always with limitations, such as on the studied population and accessibility to certain variables, including HBV viral load and the necessity of analysis of virus mutations [32,40]. The REACH-B score, for instance, took into account only patients without cirrhosis, which is an important limitation [41]. Some scores measure the risk of HCC in cirrhosis but include patients with other etiologies of liver disease, which makes it difficult to individualize the analysis [39,42].

The most recently developed and validated scores were PAGE-B and mPAGE-B, which, in addition to encompassing age and sex, included other easily accessible predictors, such as platelets and albumin [19,20,43]. In the current study, when applying these scores at the initial outpatient follow-up, it was observed that those who progressed to HCC had higher median risk values, making it possible to establish a cutoff point with good accuracy in HCC prediction. According to these results, one might infer that, in patients with mPAGE-B below 10.5 points, evolution to HCC in 5 years could be almost ruled out due to the high NPV of the score. In a study conducted by Yip et al. (2019), mPAGE-B showed relevant AUROCs at 3 and 5 years of 0.79 (0.78–0.81) and 0.8 (0.79–0.81), respectively, superior to PAGE-B [43]. On the contrary, Kirino et al. (2020) reported a slight superiority of mPAGE-B [38]. The AUROCs encountered in the present evaluation were similar to these and other reports [38,43,44], without differences between the evaluated scores. However, it is important to emphasize that the current study included patients without antiviral therapy and from an admixed population, such as the Brazilian population. Only one study evaluated PAGE-B performance in HBV patients from Brazil and none with mPAGE-B. Costa et al. (2022) evaluated the performance of PAGE-B in 978 HBV subjects from the northeast of the country, 39.5% on antiviral therapy and 18.7% with cirrhosis [21]. The authors reported an AUROC of 0.78, similar to the one in the present study, and both included nontreated patients and a close percentage of patients with cirrhosis.

It is important to note that the AUROC values may not be as clinically useful for analyzing the applicability of risk scores for HCC [45]. As the main clinical benefit of these scores is the accurate identification of patients who require or do not require HCC surveillance, the ideal indicator should be the NPV, which is naturally associated with, but not identical to, the AUROC value [45]. The NPV for both scores in the present study was high, emphasizing the PAGE-B and mPAGE-B NPV of 99.14% and 98.58%, respectively, strengthening its applicability in the studied population. Interestingly, in a cost evaluation study of the application of a tailored HCC screening based on PAGE-B risk score in HBV patients on antiviral therapy in Germany, Sprinzl et al. (2021) demonstrated a 15.51% reduction in the annual costs of surveillance [46]. This finding is even more relevant in places with limited budgets for public health services, such as the Sistema Unico de Saude (SUS) in Brazil, given the growing demands in healthcare.

This study does have some limitations. The retrospective design based on information from medical records and missing data may have interfered with the results. The lack of evaluation of HBV genotype, the evolution of transaminases, alpha-fetoprotein levels, smoking, and components of the metabolic syndrome throughout the follow-up period is also a limitation of the study. The number of included patients is limited. In addition, data on HCC staging, its therapy, and mortality could have provided additional analysis. On the other hand, this is the first study that evaluated the performance of mPAGE score in the Brazilian population.

## 5. Conclusions

In conclusion, we showed that the prevalence of HCC in this population was 6.7% at a median follow-up time of 9 years, and its occurrence was related to men, older age at HBV diagnosis, higher initial PAGE-B and mPAGE-B scores, and the presence of cirrhosis. Older age, male sex, and cirrhosis at HBV diagnosis were independently associated with HCC development. The PAGE-B and mPAGE-B scores had AUROCs above 0.79 in the prediction of HCC in these patients, with no differences between them. In addition, both scores showed high negative predictive values.

## Figures and Tables

**Table 1:** Population characteristics (*n* = 224).

Characteristics	*n* (%) or Mean ± SD
Men/women	148 (66.1%)/76 (33.9%)
Age at diagnosis (years)	38.71 ± 14.19
Systemic arterial hypertension	91 (40.6%)
Dyslipidemia	65 (29.0%)
Chronic kidney disease	55 (24.6%)
Type 2 diabetes mellitus	39 (17.4%)
Alcohol use (*n* = 221)	30 (13.6%)
HBeAg positive at diagnosis	72 (32.1%)
Cirrhosis at diagnosis (*n* = 205)	45 (21.9%)
Outpatient follow-up period (months)	133.59 ± 102.26
Hepatitis B treatment during follow-up	123/224 (54.9%)
Interferon	6/123 (4.9%)
IFN followed by nucleos(t)ide analogs	18/123 (14.6%)
Nucleos(t)ide analogs	99/123 (80.5%)
HBeAg seroclearance (*n* = 72)	40 (55.6%)
HBsAg seroclearance	33 (14.7%)
Cirrhosis at end of follow-up	86 (38.4%)
HCC development	15 (6.7%)
Follow-up period to HCC diagnosis (months)	103.13 ± 78.99
Initial PAGE-B (value)	11.58 ± 6.05
Initial mPAGE-B (value)	8.90 ± 4.16
Initial PAGE-B (stratified)	
1	78 (34.8%)
2	106 (47.3%)
3	40 (17.9%)
Initial mPAGE-B (stratified)	
1	105 (46.9%)
2	74 (33.0%)
3	45 (20.1%)

HCC: hepatocellular carcinoma; SD: standard deviation.

**Table 2:** Factors associated with HCC occurrence (*n* = 224).

Characteristics	With HCC (*n* = 15) *n* (%) or Mean ± SD	Without HCC (*n* = 209) *n* (%) or Mean ± SD	*p* Value
Men/women	14 (93.3%)/1 (6.7%)	134 (64.1%)/75 (35.9%)	0.0210 ^1^
Age at HBV diagnosis (years)	50.00 ± 12.58	37.89 ± 13.98	<0.0021 ^1^
Systemic arterial hypertension	8 (53.3%)	83 (39.7%)	0.2995
Dyslipidemia	4 (26.7%)	61 (29.2%)	1.0000
Chronic kidney disease	2 (13.3%)	53 (25.4%)	0.3701
Type 2 diabetes mellitus	4 (26.7%)	35 (16.7%)	0.3037
Alcohol use (*n* = 221)	0 (0.0%)	30 (14.6%)	0.2322
HBeAg positive at diagnosis	3 (20.0%)	69 (33.0%)	0.3971
Cirrhosis at HBV diagnosis (*n* = 205)	11 (73.3%)	34 (17.9%)	<0.0001 ^1^
Follow-up period (months)	105.79 ± 79.10	135.46 ± 103.52	0.3923
HBeAg seroclearance	1 (33.3%)	39 (56.5%)	0.5815
HBsAg seroclearance	2 (13.3%)	31 (14.8%)	1.0000
Cirrhosis at end of follow-up	15 (100.0%)	71 (34.0%)	<0.0001 ^1^
Initial PAGE-B (value)	17.07 ± 3.03	11.18 ± 6.02	0.0002 ^1^
Initial mPAGE-B (value)	12.67 ± 2.55	8.63 ± 4.12	0.0002 ^1^
Initial PAGE-B (stratification)			0.0014 ^1^
1	0 (0.0%)	78 (37.3%)	
2	8 (53.3%)	98 (46.9%)	
3	7 (46.7%)	33 (15.8%)	
Initial mPAGE-B (stratification)			0.0003 ^1^
1	1 (6.7%)	104 (49.8%)	
2	6 (40.0%)	68 (32.5%)	
3	8 (53.3%)	37 (17.7%)	

Mann–Whitney, Chi-square, and Fisher’s exact tests. HBV: hepatitis B virus; HCC: hepatocellular carcinoma; SD: standard deviation. ^1^ *p* < 0.05.

**Table 3:** Factors associated with HCC occurrence—univariate logistic regression.

Characteristics	Hazard Ratio	CI 95%	*p* Value
Men	7.879	1.036–59.933	0.0461 ^1^
Age at HBV diagnosis	1.076	1.037–1.117	0.0001 ^1^
Systemic arterial hypertension	1.346	0.487–3.722	0.5669
Dyslipidemia	0.677	0.215–2.130	0.5049
Chronic kidney disease	0.359	0.081–1.596	0.1783
Type 2 diabetes mellitus	1.484	0.472–4.666	0.4994
HBeAg negative at diagnosis	2.516	0.848–7.463	0.0962
Cirrhosis at HBV diagnosis	21.531	6.585–70.401	<0.0001 ^1^
Lack of HBsAg seroclearance	2.419	0.680–8.608	0.1727
Initial PAGE-B (value)	1.321	1.143–1.528	0.0002 ^1^
Initial mPAGE-B (value)	1.408	1.201–1.652	<0.0001 ^1^
Initial PAGE-B (stratification) (3 vs. 1 + 2)	5.870	2.107–16.352	0.0007 ^1^
Initial mPAGE-B (stratification) (3 vs. 1 + 2)	9.125	3.084–27.000	<0.0001 ^1^

HBV: hepatitis B virus; HCC: hepatocellular carcinoma; CI: confidence interval. ^1^ *p* < 0.05.

**Table 4:** Cumulative probability of hepatocellular carcinoma according to the scores.

	% (Standard Error)			
Time	3 Years	5 Years	7 Years	*p* Value
PAGE-B 1 ^1^	0.0% (0.0%)	0.0% (0.0%)	0.0% (0.0%)	
PAGE-B 2	0.0% (0.0%)	3.67% (2.08%)	5.01% (2.45%)	
PAGE-B 3	5.72% (3.93%)	13.29% (6.29%)	17.63% (7.32%)	
mPAGE-B 1	0.0% (0.0%)	0.0% (0.0%)	0.0% (0.0%)	<0.0001
mPAGE-B 2	1.45% (1.44%)	6.57% (3.19%)	6.57% (3.19%)	
mPAGE-B 3	2.70% (2.67%)	9.82% (5.45%)	18.12% (7.50%)	
Risk 1 + 2 vs. 3				
PAGE-B 1 + 2	0.0% (0.0%)	2.08% (1.19%)	2.83% (1.39%)	0.0001
PAGE-B 3	5.72% (3.93%)	13.29% (6.29%)	17.63% (7.32%)	
mPAGE-B 1 + 2	0.6% (0.6%)	2.70% (1.33%)	2.70% (1.33%)	<0.0001
mPAGE-B 3	2.70% (2.67%)	9.82% (5.45%)	18.12% (7.50%)	

Log-rank test. ^1^ It was not possible to apply the test.

**Table 5:** Performance of the initial scores in hepatocellular carcinoma prediction.

Score	AUROC (95%CI)	Cutoff	Accuracy
PAGE-B	0.7906 (0.7007–0.8805)	>14	Sensitivity: 93.33%
			Specificity: 55.50%
			NPV: 99.14% PPV: 13.08%
			PLR: 2.10% NLR: 0.12%
mPAGE-B	0.7904 (0.7026–0.8783)	>10.5	Sensitivity: 86.67%
			Specificity: 66.51%
			NPV: 98.58% PPV: 15.66%
			PLR: 2.59% NLR: 0.20%

AUROC: area under the receiver-operating characteristic; CI: confidence interval; NLR: negative likelihood ratio; NPV: negative predictive value; PLR: positive likelihood ratio; PPV: positive predictive value.

**Table 6:** Performance of the initial scores stratified by HBV therapy during follow-up.

	Score	AUROC (95%CI)	Cutoff	Accuracy
HBV therapy during follow-up	PAGE-B	0.7634 (0.6395–0.8873)	>14	Sensitivity: 100%
				Specificity: 47.32%
				NPV: 100% PPV: 15.71%
				PLR: 1.90% NLR: 0.00%
	mPAGE-B	0.7597 (0.6276–0.8919)	>11	Sensitivity: 81.82%
				Specificity: 62.50%
				NPV: 97.22% PPV: 17.65%
				PLR: 2.18% NLR: 0.29%
Absence of HBV therapy during follow-up	PAGE-B	0.8119 (0.6558–0.9679)	>17	Sensitivity: 75%
				Specificity: 83.51%
				NPV: 98.78% PPV: 15.79%
				PLR: 4.55% NLR: 0.30%
	mPAGE-B	0.8273 (0.7306–0.9240)	>11	Sensitivity: 100%
				Specificity: 71.13%
				NPV: 100% PPV: 12.50%
				PLR: 3.46% NLR: 0.00%

AUROC: area under the receiver-operating characteristic; HBV: hepatitis B virus; CI: confidence interval; NLR: negative likelihood ratio; NPV: negative predictive value; PLR: positive likelihood ratio; PPV: positive predictive value.

## Data Availability

The raw data required to reproduce these results are available from the corresponding author upon reasonable request.
